# CD300a inhibits CD16-mediated NK cell effector functions in HIV-1-infected patients

**DOI:** 10.1038/s41423-019-0275-4

**Published:** 2019-08-29

**Authors:** Joana Vitallé, Iñigo Terrén, Ane Orrantia, Raquel Pérez-Garay, Francesc Vidal, José A. Iribarren, Carmen Rodríguez, Ana M. López Lirola, Enrique Bernal, Olatz Zenarruzabeitia, Francisco Borrego

**Affiliations:** 1 Immunopathology Group, Biocruces Bizkaia Health Research Institute, Barakaldo, Spain; 2 Hospital Universitari de Tarragona Joan XXIII, IISPV, Universitat Rovira i Virgili, Tarragona, Spain; 3 Servicio de Enfermedades Infecciosas, Hospital Universitario Donostia, Instituto de Investigación Sanitaria Biodonostia, Donostia-San Sebastián, Spain; 4 Centro Sanitario Sandoval, Hospital Clínico San Carlos, Instituto de Investigación Sanitaria San Carlos, Madrid, Spain; 5 Hospital Universitario de Canarias, Santa Cruz de Tenerife, Spain; 6 Department of Clinical Medicine, Universidad de Murcia and Hospital General Universitario Reina Sofía, Murcia, Spain; 7 Ikerbasque, Basque Foundation for Science, Bilbao, Spain

**Keywords:** NK cells, HIV infections, Innate lymphoid cells

Natural killer (NK) cell-mediated antibody-dependent cellular cytotoxicity (ADCC) through CD16 plays a critical role in anti-human immunodeficiency virus (HIV) responses.^1–3^ CD300a is a surface receptor highly expressed on NK cells that has the capacity to inhibit NK cell-mediated cytotoxicity in healthy donors.^4^ The CD300a molecule has been related to several viral infections and is able to diminish the NK cell killing of pseudorabies-infected cells through interactions with its ligands phosphatidylserine and phosphatidylethanolamine.^5^ In addition, CD300a expression on B and CD4+ T lymphocytes is altered during HIV-1 infection, and combined antiretroviral therapy (cART) does not restore nonpathological expression levels.^5,6^ However, the expression and function of CD300a on NK cells during HIV-1 infection is still unknown. We have determined the surface expression of CD300a on different NK cell subsets and the capacity of this receptor to inhibit CD16-induced NK cell effector functions in healthy and HIV-1 infected individuals.

As HIV-1 infection modulates CD300a expression on some immune cells,^5,6^ we first analyzed the expression of the CD300a receptor on different NK cell subpopulations from healthy donors, untreated HIV-1-infected subjects, and patients on cART by flow cytometry. The samples were provided by the HIV BioBank integrated in the Spanish AIDS Research Network (RIS) (see Supplementary Material). Clinical data from HIV-1-infected patients are shown in Table S1. Three different NK cell subsets were studied: CD56^bright^ (CD56^++^NKp80^+^), CD56^dim^ (CD56^+^NKp80^+^), and CD56^neg^ (CD56^−^NKp80^+^) (Fig. S1). When we examined the CD300a expression, no significant differences were observed between the groups, with the exception of CD56^neg^ NK cells, which displayed a higher frequency of CD300a+ cells in untreated HIV-1 infected patients (Fig. S2). Ongoing HIV replication induces the expansion of a dysfunctional CD56^neg^ NK cell subset^1,7,8^ (Fig. S1). Thus, we suggest that the overexpression of the CD300a inhibitory receptor on the CD56^neg^ NK cell subset may contribute to the dysfunctionality observed in this expanded population in HIV-1 infected patients, which is partially restored with cART.

We also examined CD300a expression on different NK cell subsets selected according to the expression of NKG2A, NKG2C, CD57, and NKp46 (Fig. S3). These receptors are altered during HIV-1 infection,^7,8^ and some of them are commonly used to distinguish NK cell maturation stages.^9^ In general, we observed that different CD300a expression levels were associated with the expression of these markers in all subjects (Fig. S4). Specifically, higher CD300a expression was found on NKG2A+ CD56^dim^ NK cells, while CD57+ cells displayed lower CD300a expression levels (Fig. S4), indicating that CD300a is more expressed on immature CD56^dim^ NK cells, a cell subset that is significantly decreased in HIV-1 infected patients.^3,7^

NK cells express the FcγRIIIA (CD16) surface receptor, which is responsible for ADCC.^1,2^ To investigate the capacity of CD300a to inhibit CD16-mediated NK cell activation in HIV-1-infected patients, we performed a redirected lysis assay (Fig. S5). We cocultured NK cells with the Fc receptor-bearing cell line P815. CD16 and CD300a from NK cells were triggered with specific mAbs, and the MOPC-21 isotype control was utilized as a negative control (Fig. S5). To study NK cell effector functions, we determined the percentage of NK cells positive for the degranulation marker CD107a, the cytokines interferon (IFN)γ and tumor necrosis factor (TNF), and the chemokine macrophage inflammatory protein (MIP)-1β utilizing flow cytometry-based procedures (see Supplementary Material).

In agreement with the literature,^1,3,7^ the CD56^dim^ NK cell subset displayed the highest response to CD16-mediated stimulation, and NK cell effector functions were significantly diminished in HIV-1 infected patients (Fig. S6). Very importantly, we observed that all NK cell subsets from the three groups exhibited lower effector functions after the CD16-mediated stimulation and cross-linking of CD300a with specific mAbs (Fig. 1a, b). Moreover, when we compared the degranulation and MIP-1β production by different NK cell subsets, we observed that CD56^bright^ cells were the most inhibited subset after CD300a cross-linking in all donors (Fig. S7), consistent with the higher CD300a expression in this NK cell subpopulation (Fig. S2). Finally, we observed a higher CD300a-mediated inhibition of degranulation and MIP-1β production by CD56^bright^ and CD56^dim^ NK cells from HIV-1 infected patients, particularly from those who were under cART (Fig. 1c).

**Fig. 1:**
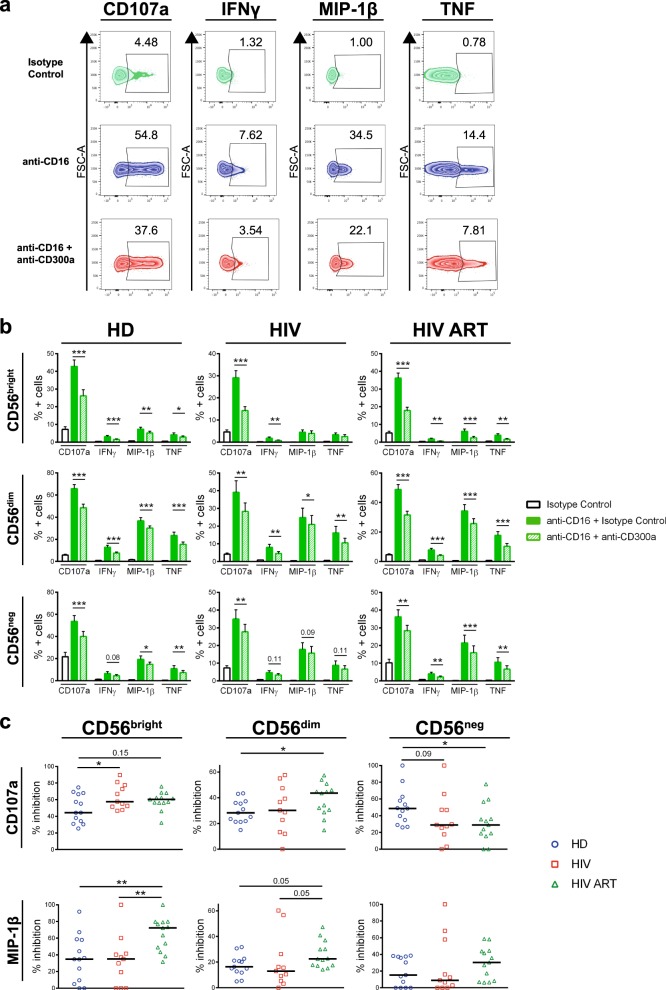
Degranulation and cytokine production by NK cells in response to the cocrosslinking of CD16 and CD300a. **a** Zebra plots showing the percentage of CD56^dim^ NK cells positive for CD107a, IFNγ, MIP-1β, and TNF from a representative untreated HIV-1 infected patient after stimulation with isotype control, anti-CD16 plus isotype control, or anti-CD16 plus anti-CD300a mAbs. **b** Bar graphs showing the percentage of CD56^bright^, CD56^dim^, and CD56^neg^ NK cells positive for CD107a, IFNγ, MIP-1β, and TNF from healthy donors (HD), untreated HIV-1 infected subjects (HIV), and subjects under cART (HIV ART) after stimulation with isotype control, anti-CD16 plus isotype control, or anti-CD16 plus anti-CD300a mAbs. The mean with the SEM is represented. **c** Dot plots showing the percentage of CD300a-mediated inhibition of degranulation (CD107a) and MIP-1β production by CD56^bright^, CD56^dim^, and CD56^neg^ NK cells, comparing HD, HIV, and HIV ART patients. Each dot represents a subject, and the median is shown. *p < 0.05, **p < 0.01, ***p < 0.001

ADCC has been demonstrated as an important factor for the long-term control of HIV-1 infection that subsequently results in better disease prognosis.^1–3,7^ Furthermore, the relevance of ADCC in new anti-HIV therapies has been emphasized with the introduction of broadly neutralizing antibodies.^2,3^ Nevertheless, decreased HIV-specific effector antibody responses have been found in HIV-1-infected individuals, including those receiving cART.^7,10^ Our results suggest that CD300a might decrease ADCC-mediated NK cell killing of HIV-infected cells by inhibiting degranulation and cytokine and chemokine production by NK cells. Previous findings have indicated that successful cART is not enough to achieve effective antibody-mediated HIV-1 control,^7,10^ highlighting the importance of our study in the search for new strategies to achieve more effective ADCC in HIV-1-infected patients. Therefore, similar to other therapeutic strategies in which NKG2A and KIR inhibitory receptors are targeted with monalizumab and lirilumab, respectively, targeting CD300a could represent a new strategy to improve NK cell functions. Nonetheless, further experiments are required to confirm our hypothesis.

## Supplementary information

Supplemental Material
