# Supplementary material for: CD300a inhibits CD16-mediated NK cell effector functions in HIV-1-infected patients
Source: Cell Mol Immunol. 2019 Aug 29;16(12):940–2. doi: 10.1038/s41423-019-0275-4 (PMC6884514; doi:10.1038/s41423-019-0275-4)
Supplement: Supplementary file 1 — Supplemental Material [file 41423_2019_275_MOESM1_ESM.pdf]

Supplementary Information

CD300a inhibits CD16-mediated NK cell effector functions in HIV-1 infected patients

Joana Vitallé, Iñigo Terrén, Ane Orrantia, Raquel Pérez-Garay, Francesc Vidal, José A. Iribarren, Carmen Rodríguez, Ana M. López Lirola, Enrique Bernal, Olatz Zenarruzabeitia, Francisco Borrego.

Donors, Materials and Methods

Subjects and Samples

For this study, cryopreserved peripheral blood mononuclear cells (PBMCs) and plasma from healthy donors (n = 13), cART naïve HIV-1 infected subjects (n = 14) and patients under cART (n = 13) were kindly provided by the HIV BioBank integrated in the Spanish AIDS Research Network (RIS). Samples were processed following current procedures and frozen immediately after their reception. All subjects provided written and signed informed consent in accordance with the Declaration of Helsinki. Institutional and Ethical Review Board approvals were obtained from all healthy donors and patients. The study was approved by the Basque Ethics Committee for Clinical Research (PI2014017 and PI2013108).

All HIV-1 infected patients were asymptomatic when the sample was collected, were not co-infected with hepatitis C virus (HCV), had more than 200 CD4+ T cells/mm^3 and they had never been diagnosed with AIDS. Untreated HIV-1 infected subjects had detectable viremia (>10,000 HIV-RNA copies/ml) and they had never been treated with cART, while patients under cART had undetectable viremia and had been treated with cART at least for 6 months. Clinical data of HIV-1 infected patients were obtained from the RIS database, which are shown in Table S1 (see below).

Antibodies

To perform flow cytometry-based experiments, the following mouse anti-human mAbs were utilized: BV421 anti-CD56 (clone NCAM 16.2), BV510 anti-CD3 (clone UCHT1), BV510 anti-CD14 (clone MϕP9), FITC anti-MIP-1β (clone D21-1351),

PerCP-Cy5.5 anti-IFNγ (clone B27), BV421 anti-CD107a (clone H4A3) and unconjugated anti-CD16 (clone 3G8) from BD Biosciences; PE anti-CD56 (clone MEM-188), PerCP-Cy5.5 anti-CD57 (clone HNK-1), APC anti-NKp46 (clone 9E2), APC anti-TNFα (clone Mab11) and unconjugated IgG isotype control (clone MOPC-21) from Biolegend; PE anti-CD300a (clone E59.126) and APC anti-NKG2A (clone Z199) from Beckman Coulter; PE-Vio770 anti-NKp80 (clone 4A4.D10) from Miltenyi Biotec; PerCP anti-NKG2C (clone 134591) from R&D Systems and unconjugated anti-CD300a (clone MEM-260) from Invitrogen.

Extracellular staining

Frozen PBMCs from healthy donors and HIV-1 infected patients were thawed at 37℃ and washed two times with R10 medium by spinning down the cells at 1,500 rpm for 5 min at room temperature. R10 medium was composed of RPMI 1640 containing GlutaMAX (Thermo Fisher Scientific), 10% of Fetal Bovine Serum (Hyclone) and 1% of penicillin/streptomycin (Thermo Fisher Scientific). Then, PBMCs were incubated in a 6 well plate in R10 at 37℃ and 5% CO_2 overnight at a concentration of 2 x 10^6 cells/ml. After the resting period, cells were washed twice with PBS containing 2.5% of Bovine Serum Albumin (BSA) at 4℃. For the phenotypical characterization of NK cells, PBMCs were first stained with the 405 nm LIVE/DEAD™ Fixable Aqua Dead Cell Stain Kit (Invitrogen) following the manufacturer's protocol, for the detection of dead cells. Afterwards, the extracellular staining was carried out by adding fluorochrome-conjugated mAbs for the detection of NK cell subsets and NK cells surface receptors. In this step, cells were incubated with the mAbs at 4℃ during 30 min in the dark. Stained PBMCs were washed again with PBS 2.5% BSA and fixed with 200µl of 4% paraformaldehyde (Sigma-Aldrich) in PBS for 15 min at 4℃. Finally,

200µl of PBS were added and sample acquisition was done in a FACS Canto II flow cytometer (BD Biosciences).

Redirected lysis assay and intracellular cytokine staining

Cryopreserved PBMCs were thawed and maintained in resting conditions overnight as explained above. For the NK cell-mediated redirected lysis assay, PBMCs were cultured in R10 medium in a U-bottom 96 well plate along with the mouse cell line P815 at a Effector:Target (E:T) ratio of 1:1, in a final concentration of 1 x 10^6 cells/200µl per well. Afterwards, the anti-CD107a mAb and GolgiStop (monensin) and GolgiPlug (brefeldin A) protein transport inhibitors were added to the cell culture according to the manufacturer's protocol (BD Biosciences). Both PBMCs and P815 cells were incubated for 6 h at 37℃ in the presence of mAbs. Three different conditions were studied: a) 2.5µg/ml of MOPC-21 isotype control; b) 2.5µg/ml of anti-CD16 and 10µg/ml of MOPC-21 isotype control; and c) 2.5µg/ml of anti-CD16 and 10µg/ml of anti-CD300a (Figure S5). After the incubation period, the intracellular cytokine staining was carried out. For that, cells were stained with LIVE/DEAD reagent to detect dead cells and with fluorochrome-conjugated mAbs for the identification of NK cell subsets as explained above. Then, cells were fixed and permeabilized using Cytofix/Cytoperm Plus Kit (BD Biosciences) following manufacturer's protocol and were incubated for 30 min at 4℃ in the dark with several fluorochrome-conjugated mAbs for the detection of intracellular cytokines. Stained cells were washed again and were resuspended in 350 µl of PBS. Lastly, samples were acquired with the FACS Canto II flow cytometer.

Laboratory methods

Anti-CMV IgM and IgG levels from plasma belonged to HIV-1 infected patients were determined using a IMMUNITE/IMMUNITE 1000 and IMMUNITE/IMMUNITE 2000 assay (Siemens), following manufacturers protocol. Immunonephelometric assays using latex particles were carried out in order to measure plasma levels of C-reactive protein (CRP) and β2-microglobulin (B2M) from HIV-1 infected patients (N Latex CRP mono and N Latex-β_2-Microglobulina, from Siemens), also following the instructions from the manufacturer.

Statistics

Flow cytometry data were analyzed utilizing FlowJo software (version 10.0.7) and graphical representation and statistical analysis were done with GraphPad Prism software (version 6.01). In order to determine the distribution of the data, D'Agostino & Pearson normality test was performed. Our data were not normally distributed, and therefore non-parametric tests were applied for the statistical analysis. For the comparison between healthy donors, untreated HIV-1 infected subjects and patients under cART, the unpaired Mann-Whitney test was utilized. The Wilcoxon matched-pairs test was applied to study the differences between cell subsets and different stimulation conditions in the functional assays. To calculate the percentage of CD300a-mediated inhibition of NK cell effector functions, the following formula was used:

$$\%\ \text{inhibition} = \left(\frac{(\alpha CD16 - isotype)}{(\alpha CD16 - isotype)} - \frac{(\alpha CD16\ \&\ \alpha CD300a - isotype)}{(\alpha CD16\ - isotype)}\right) \times 100.$$

Table S1. Clinical data of naïve and under cART HIV-1 infected patients.

	**Naïve HIV-1 patients**		**HIV-1 patients on cART**	
	**Median**	**Range (min-max)**	**Median**	**Range (min-max)**
**Sex**	Male: n=14<br>Female: n=0	-	Male: n=12<br>Female: n=1	-
**Age (years)**	27.5	(22 – 49)	39	(25 – 47)
**cART (years)**	-	-	1	(1 – 3)
**Viral load (RNA copies/ml)**	29,600	(11,735 – 125,892)	<20	-
**CD4+ T cells/mm^3**	501.5	(33 – 915)	612	(344 – 1043)
**CMV**	Negative: n=0<br>Positive: n=14	-	Negative: n=3<br>Positive: n=10	-
**B2M (mg/l)**	2.2	(0.99 – 3.05)	2.2	(1.47– 3.58)
**CRP (mg/l)**	1.0	(0.15 – 7.22)	1.8	(0.17 – 8.43)

Supplementary Figures

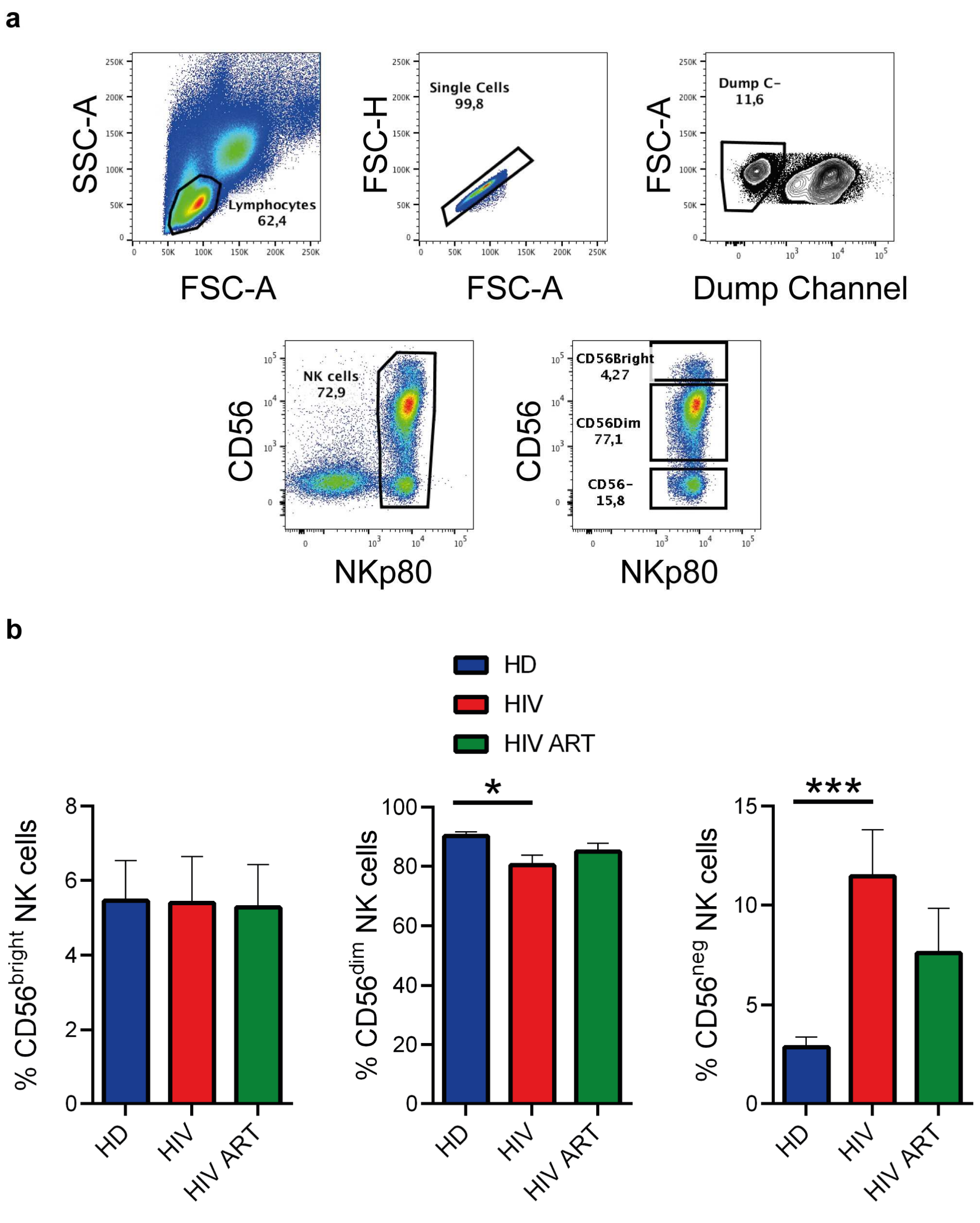

Fig. S1. NK cell subsets from healthy donors and HIV-1 infected patients. (**a**) Pseudocolor and contour plots representing the gating strategy utilized in this study. Data from a representative untreated HIV-1 infected patient is shown. Lymphocytes were electronically gated based on their forward and side scatter parameters and then single cells were selected. To identify NK cells, the population negative for the dump

channel (viability, CD3 and CD14) was selected and then three NK cell subsets were identified: CD56^{bright} (CD56++NKp80+), CD56^{dim} (CD56+NKp80+) and CD56^{neg} (CD56-NKp80+). (**b**) Bar graphs showing the percentage of each NK cell subpopulation from healthy donors (HD), untreated HIV-1 infected subjects (HIV) and patients under cART (HIV ART). The mean with the standard error of the mean (SEM) is represented. *p<0.05, *** p<0.001.

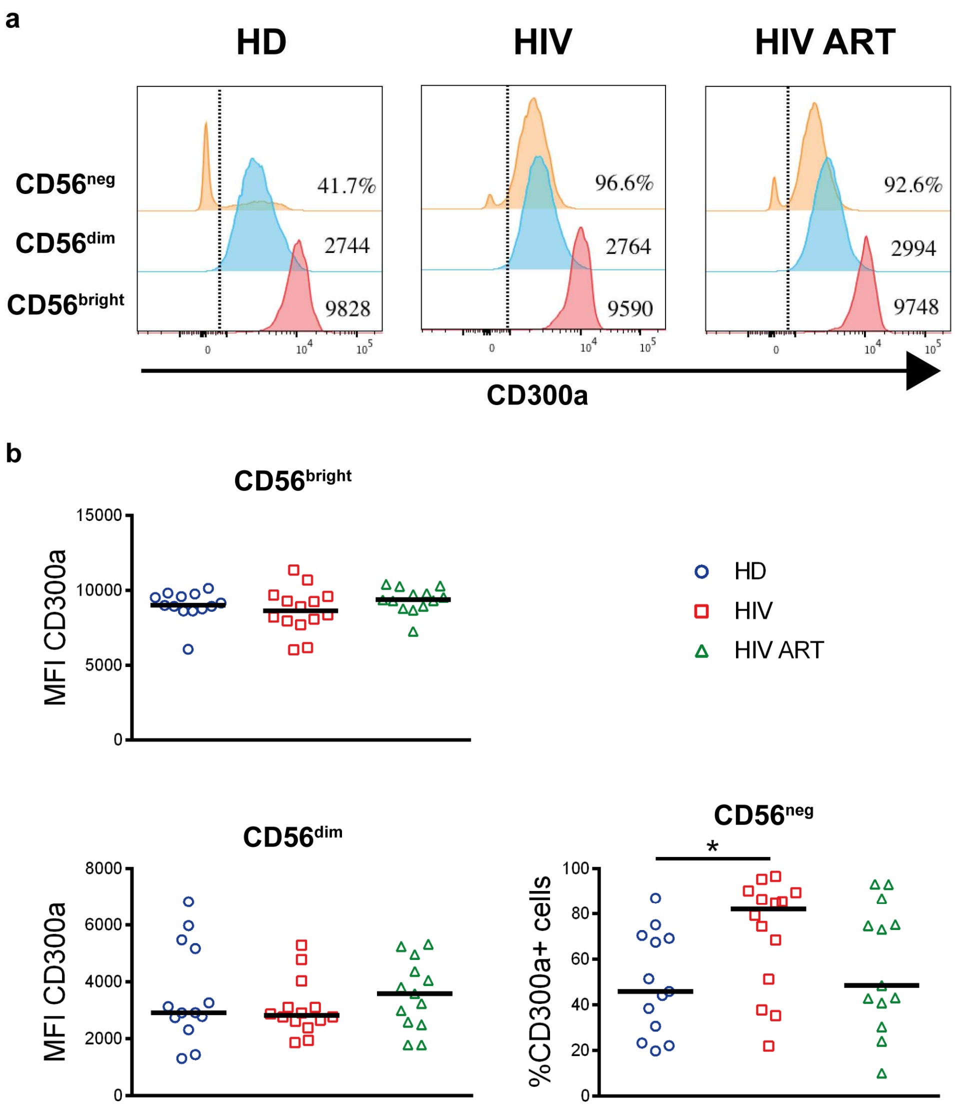

Fig. S2. CD300a expression on NK cell subpopulations from healthy donors and HIV-1 infected patients. (**a**) Histograms showing the percentage of CD300a+ cells on $CD56^{neg}$ NK cells and the median fluorescence intensity (MFI) of CD300a on $CD56^{bright}$ and $CD56^{dim}$ NK cells. Data from a representative a healthy donor (HD), an untreated HIV-1 infected subject (HIV) and a patient on cART (HIV ART) are shown. (**b**) Dot plots showing the MFI of CD300a on $CD56^{bright}$ and $CD56^{dim}$ NK cells, and the percentage of CD300a+ cells on $CD56^{neg}$ NK cells, from HD, HIV and HIV ART patients. Each dot represents a subject and the median is shown. $^{*}p<0.05$.

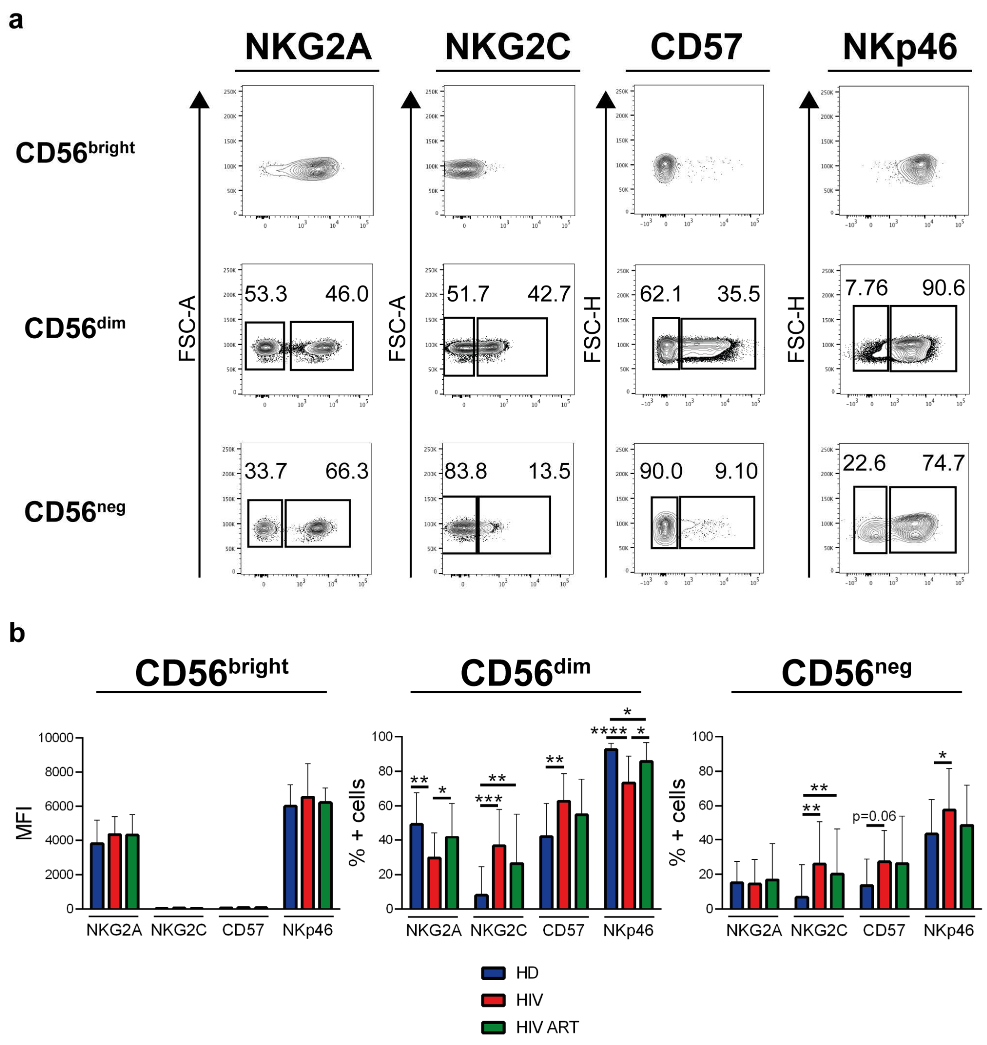

Fig. S3. Expression of NK cell surface receptors on NK cells from healthy donors and HIV-1 infected patients. (**a**) Contour plots representing the expression of NKG2A, NKG2C, CD57 and NKp46 on CD56^{bright} NK cells and the percentage of NKG2A, NKG2C, CD57 and NKp46 expressing cells within CD56^{dim} and CD56^{neg} NK cells from a representative untreated HIV-1 infected patient. (**b**) Bar graphs showing the MFI of NKG2A, NKG2C, CD57 and NKp46 on CD56^{bright} NK cells and the percentage of cells positive for NKG2A, NKG2C, CD57 and NKp46 within CD56^{dim} and CD56^{neg} NK cells from healthy donors (HD), untreated HIV-1 infected subjects (HIV) and patients under cART (HIV ART). The mean with the standard deviation (SD) is represented. *$p<0.05$, ** $p<0.01$, *** $p<0.001$, **** $p<0.0001$.

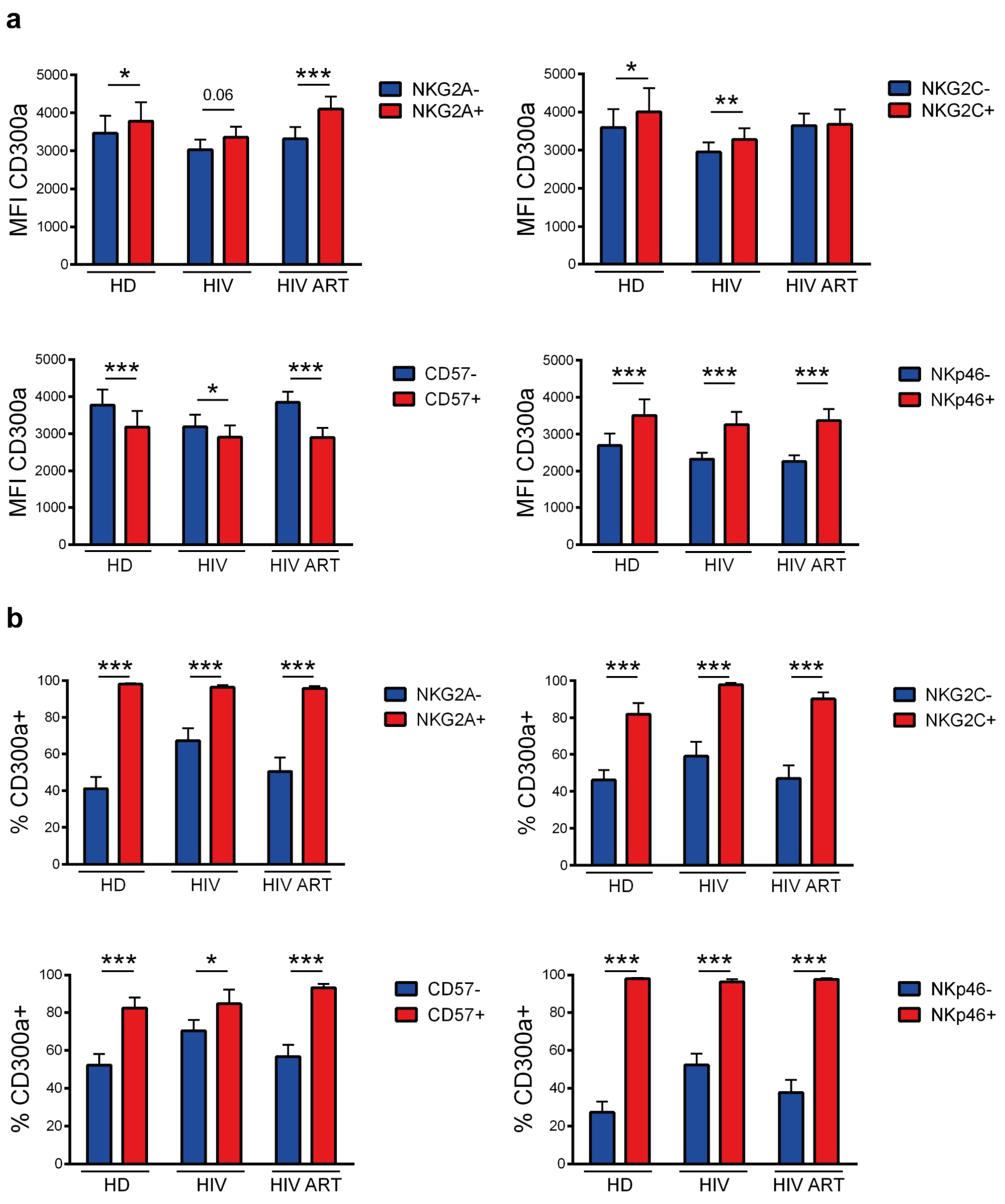

Fig. S4. CD300a expression on NK cell subsets according to the expression of NKG2A, NKG2C, CD57 and NKp46 receptors from healthy donors and HIV-1 infected patients. (**a**) Bar graphs showing the MFI of CD300a on CD56^{dim} NK cells from healthy donors (HD), cART naïve HIV-1 infected subjects (HIV) and patients on cART (HIV ART). (**b**) Bar graphs representing the percentage of CD300a+ cells on CD56^{neg} NK cells from HD, HIV and HIV ART patients. The mean with the SEM is represented. *p<0.05, ** p<0.01, *** p<0.001.

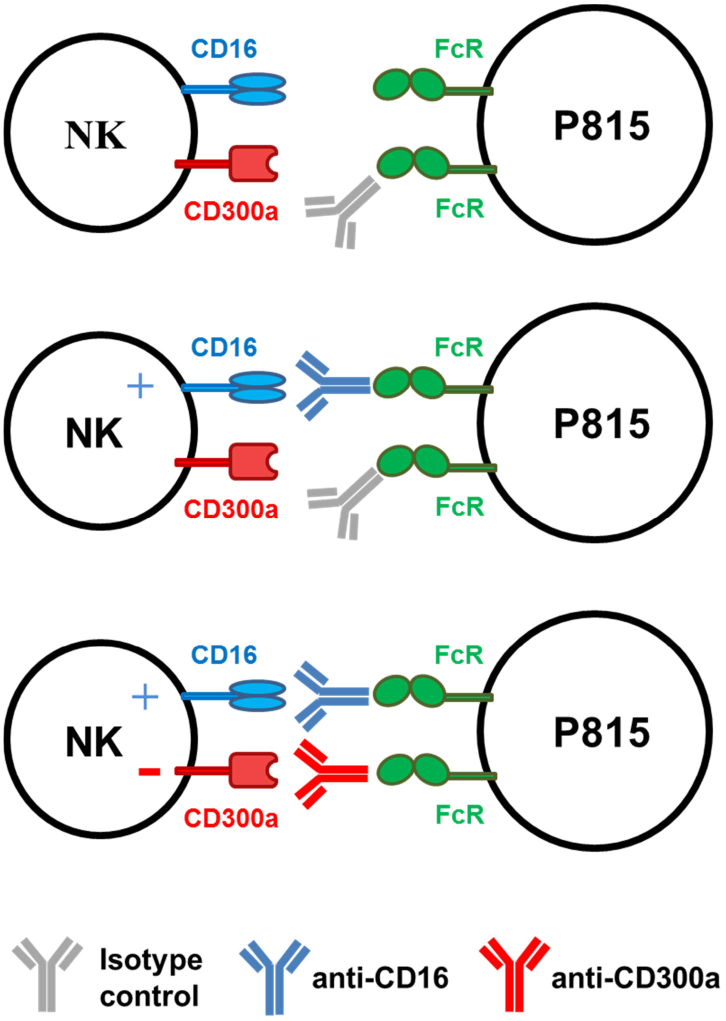

Fig. S5. Schematic representation of redirected lysis assay. Redirected lysis assay was carried out by co-culturing NK cells with the FcR-bearing cell line P815. This assay included three experimental conditions, in which different mAbs were added: isotype control (top), anti-CD16 plus isotype control (middle) and anti-CD16 plus anti-CD300a (below). The isotype control only binds FcRs from P815 cells, and therefore it does not transmit any signal on NK cells. NK cell activation is induced by triggering the CD16 receptor with a specific mAb, whose Fc portion binds FcR on P815 cells. The crosslinking of CD300a with a specific mAb, which is also able to bind FcR on P815, induces inhibitory signals on NK cells.

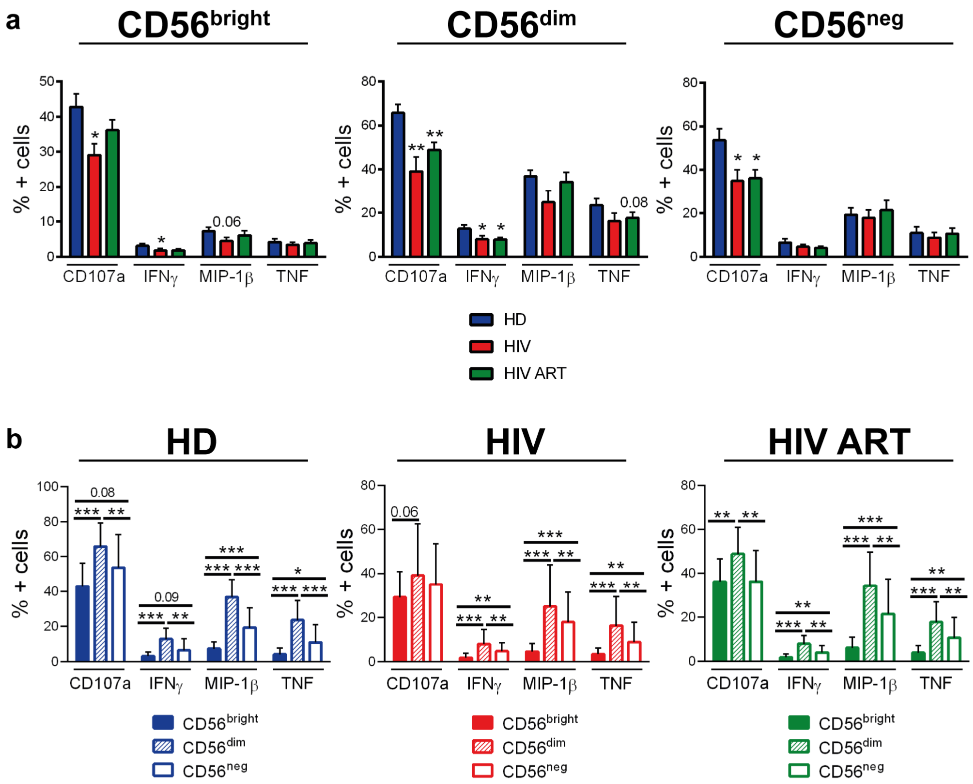

Fig. S6. CD16-mediated NK cell degranulation and cytokine production. (a) Bar graphs showing the percentage of $CD56^{bright}$ (left panel), $CD56^{dim}$ (middle panel) and $CD56^{neg}$ (right panel) NK cells positive for CD107a, IFNγ, MIP-1β and TNF after the stimulation through the CD16 receptor, comparing healthy donors (HD), untreated HIV-1 infected subjects (HIV) and patients under cART (HIV ART). Statistical analyses are made only between HD with HIV and HIV ART. The mean with the SEM is represented. (b) Bar graphs showing the percentage of cells positive for CD107a, IFNγ, MIP-1β and TNF, within $CD56^{bright}$ vs $CD56^{dim}$ vs $CD56^{neg}$ NK cells after the stimulation through the CD16 receptor, in HD (left panel), HIV (middle panel) and patients HIV ART (right panel). The mean with the SD is represented. *$p<0.05$, ** $p<0.01$, *** $p<0.001$.

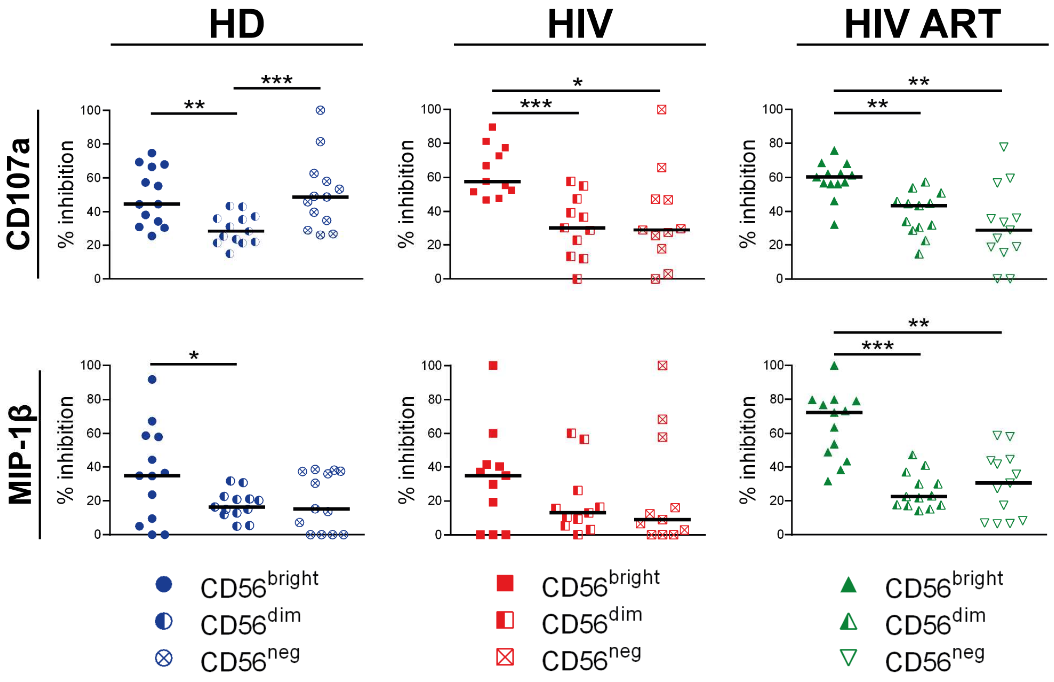

Fig. S7. Percentage of CD300a-mediated inhibition of the degranulation and MIP-1β production by NK cells. Dot plots showing the percentage of CD300a-mediated inhibition of degranulation (CD107a) and MIP-1β production by CD56^{bright} vs CD56^{dim} vs CD56^{neg} NK cells, from healthy donors (HD), untreated HIV-1 infected subjects (HIV) and patients under cART (HIV ART). Each dot represents a subject and the median is shown. *$p<0.05$, ** $p<0.01$, *** $p<0.001$.
